# Comparison of Diastolic Function Parameters After Alcohol Septal Ablation and Mavacamten Therapy in Obstructive Hypertrophic Cardiomyopathy

**DOI:** 10.3390/jcdd13010016

**Published:** 2025-12-29

**Authors:** Danish Saleh, Ellis Y. Kim, Kifah Hussain, Ashraf Samhan, Meilynn Shi, Zhiying Meng, Elizabeth Schormann, Parmeen Bindra, Baljash Cheema, Dominic E. Fullenkamp, Abigail S. Baldridge, Jyothy J. Puthumana, Vera H. Rigolin, Paul C. Cremer, James D. Flaherty, Lubna Choudhury

**Affiliations:** 1 Department of Medicine, Division of Cardiology, Feinberg School of Medicine, Northwestern University, Chicago, IL 60611, USA; 2 Department of Medicine, Division of Cardiology, School of Medicine and Public Health, University of Wisconsin—Madison, Madison, WI 53791, USA; 3 Bluhm Cardiovascular Institute, Northwestern University Feinberg School of Medicine, Chicago, IL 60611, USA; 4 The Hypertrophic Cardiomyopathy Program at the Bluhm Cardiovascular Institute, Chicago, IL 60611, USA

**Keywords:** hypertrophic cardiomyopathy, mavacamten, alcohol septal ablation, diastolic function, echocardiography

## Abstract

Cardiac myosin inhibitors have been shown to improve diastolic function in patients with obstructive hypertrophic cardiomyopathy (HCM). Comparative studies to evaluate the diastolic effects of mavacamten versus alcohol septal ablation (ASA) have yet to be examined. In this single-center retrospective analysis, we compared echocardiographic parameters of diastolic function in adult patients with obstructive HCM treated with mavacamten (*n* = 23) or ASA (*n* = 22). Baseline imaging was obtained prior to therapy, and follow-up imaging was obtained five months after ASA and or initiation of mavacamten. Left-sided filling pressures (E/e’) improved with both ASA (18.6 versus 15.3, *p* < 0.001) and mavacamten (17.4 versus 13.5, *p* = 0.01). Among patients who underwent ASA, mitral annular tissue velocity (e’) was increased at the lateral annulus (6.0 versus 6.1, *p* = 0.02) with a trend to improvement at the septum (4.0 versus 5.0, *p* = 0.14). Similarly, among patients treated with mavacamten, septal e’ was increased (6.0 versus 6.7, *p* < 0.01) and a trended improvement was observed for the lateral e’ (5.7 versus 7.0, *p* = 0.06). Mavacamten therapy was also associated with an improvement in the LA volume index (45.6 versus 34.5, *p* < 0.001). Patients treated with ASA were older, more likely to have used tobacco, and had greater limitation in functional status. In this retrospective analysis, ASA and mavacamten were similarly associated with improvements in echocardiographic parameters of diastolic function and left-sided filling pressures, though mavacamten had a more discernible effect on the left-atrial volume index. Larger studies are required to further characterize the relative efficacy of the two therapeutic modalities.

## 1. Introduction

Hypertrophic cardiomyopathy (HCM) is the most common familial cardiomyopathy and is inherited in an autosomal dominant fashion [1,2]. The prevalence of HCM is estimated to be between 1:500 and 1:200 in the general population and impacts more than 20 million people worldwide [3,4]. Mutations associated with HCM disrupt sarcomere formation, causing myofibril disarray and cellular hypertrophy as determined histologically [5]. Structurally, HCM manifests with pathological thickness of the myocardium, tissue scarring, and ventricular stiffening, which are associated with the clinical features of heart failure [5].

Heart failure in the setting of HCM may be observed as a function of obstructive or non-obstructive left-ventricular outflow tract (LVOT) hemodynamics [6]. Two-thirds of patients with HCM exhibit obstructive hemodynamics. Heart failure associated with obstructive physiology is attributed to a narrowed outflow tract and steep pressure gradients (>30 mmHg at rest and >50 mmHg with provocation) across the LVOT that result from hypercontractility, ventricular thickness, and aberrations in the mitral valve apparatus [7,8,9,10]. Conversely, heart failure associated with non-obstructive physiology primarily results from diastolic heart disease ascribed to pathologic myocardial thickness, a small ventricular cavity, and impaired myocardial relaxation.

Pharmacologic agents that improve left-ventricular filling and reduce myocardial contractility, including beta blockers, calcium channel blockers, and disopyramide, have historically served as first-line therapies in the treatment of obstructive HCM [1,2]. Septal-reduction therapies (SRTs), including alcohol septal ablation (ASA) and surgical myectomy, are considered in patients who have been either unable to tolerate or are refractory to first-line medical therapies [1,2,11,12,13]. ASA is a catheter-based approach aimed at thinning the interventricular septum (IVS) by delivering alcohol into the proximal septal perforators to induce a controlled myocardial infarction within the septum [14]. Historically, ASA is the preferred strategy for SRT in patients who are not candidates for surgical myectomy [2,15].

Myosin inhibitors have emerged as a novel therapeutic tool to dampen myocardial contractility and relieve LVOT obstruction in HCM [2,16,17,18,19,20,21]. These small molecules inhibit myosin-head-associated ATPase activity and thereby reduce actin–myosin cross-bridging within the sarcomere [16,17]. Mavacamten is the first commercially available myosin inhibitor that has been shown to improve cardiac hemodynamics and functional performance in patients with obstructive HCM [18,19,22]. Emerging evidence suggests cardiac myosin inhibitors may directly improve echocardiographic parameters of diastolic function [22,23,24]. We have previously observed that septal myectomy is associated with improvements in diastolic function in patients with HCM. Additionally, we recently reported that SRT with ASA yields comparable improvements in obstructive hemodynamics and patient functional status when compared to mavacamten [23]. In this follow-up study, we sought to examine how mavacamten compares with ASA in modifying parameters of diastolic function.

## 2. Materials and Methods

### 2.1. Study Population and End Points

**Figure 1:**
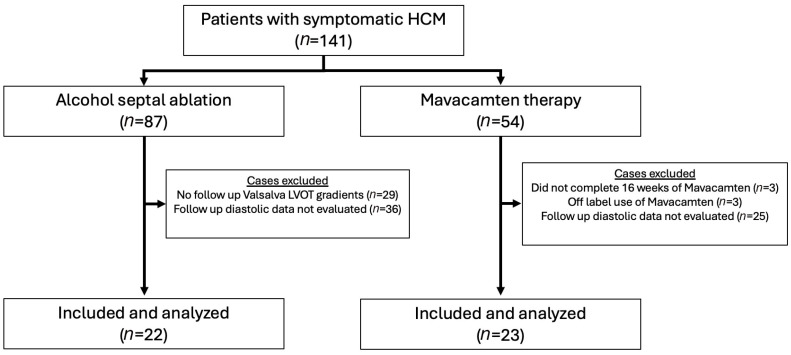
CONSORT diagram illustrating patient selection for the study cohorts.

Patients of the Bluhm Cardiovascular Institute (BCVI) of Northwestern Memorial Hospital in Chicago, IL, managed for HCM from July 2012 to May 2024 were sampled for this study. Patients who had undergone ASA (*n* = 87) were identified through individual chart review. Patients currently or previously treated with mavacamten were identified through the Risk Evaluation and Mitigation Strategy (REMS) enrollment at Northwestern (*n* = 54) (Figure 1). Clinical and echocardiographic data were collected and reported for individuals at baseline and at 5 months (average 5.1 months; interquartile range (IQR) 3.5–6.0 months) following ASA or initiation of mavacamten, respectively. Mavacamten dosing was coordinated as described in the prescribing information literature issued by Bristol Myers Squibb. In the ASA arm, 29 patients were excluded because follow-up echocardiograms with assessment of Valsalva-induced LVOT gradients were not available between 3 and 12 months of the procedure. Among the residual individuals, an additional 36 individuals were excluded because diastolic parameters were not obtained during follow-up imaging. In the mavacamten arm, six individuals were excluded. Three of these individuals were excluded because they had not completed at least 16 weeks of follow-up. One patient in the mavacamten cohort was excluded because dosing was initiated at a 2.5 mg dose, which is outside the parameters of the REMS dosing protocol, and two others were excluded because pre-treatment Valsalva outflow tract gradients were less than 50 mmHg. Among the residual individuals, an additional 25 were excluded because diastolic parameters were not obtained during follow-up imaging. NYHA class was determined by chart review and clinical documentation. Left-ventricular dysfunction requiring discontinuation of mavacamten therapy (LVEF < 50%) was not observed within 36 weeks of initiating therapy in any participants. Baseline characteristics are expressed as the mean and standard deviation for continuous variables and frequency and percent (%) for categorical variables.

### 2.2. Imaging Assessment

Echocardiograms were performed in accordance with contemporary American Society of Echocardiography (ASE) guidelines [25]. LVOT gradients, mitral inflow (E and A), tissue Doppler annular velocities (E’), and left-atrial volume indices were assessed in accordance with ASE guidelines [26].

### 2.3. Statistics

Baseline and functional characteristics were reported as mean (standard deviation), median (interquartile range), or number (%). Demographic characteristics and clinical outcomes between treatment groups were compared using a two-sample *t*-test, the Chi-squared test, or Fisher’s exact test as appropriate. Differences within each treatment group, between baseline and follow-up functional characteristics, were compared using the paired Student *t*-test or Wilcoxon signed rank test as appropriate. Statistical significance was defined as *p* < 0.05. Statistical analysis was performed using SAS (v9.4, Cary NC, USA) and R version 4.2.2. Institutional Review Board approval at Northwestern University was obtained before the initiation of this study.

## 3. Results

The study groups comprised patients diagnosed with obstructive HCM who underwent ASA (*n* = 22) compared with patients who initiated treatment with mavacamten (*n* = 23). The two groups were similar with respect to multiple demographic criteria and elements of the medical history, including body mass index (BMI), sex distribution, history of hypertension, history of atrial fibrillation, presence of an in situ implantable cardioverter defibrillator (ICD), and prior use of beta blockers (*p* > 0.05 for all) (Table 1). Patients in the ASA group were older (76 versus 61 years, *p* < 0.001) and were more likely to have used tobacco (77% versus 13%, *p* < 0.001). Patients in the ASA arm were more likely to have been treated with non-dihydropyridine calcium channel blockers (*p* = 0.002) and disopyramide (*p* = 0.047) and had a poorer functional status such that 71.4% described NYHA Class III limitations compared to 30.4% in the mavacamten arm (*p* = 0.007). There were three patients in the mavacamten arm and one patient in the ASA arm who had previously undergone septal reduction therapy. Similar observations were made within the larger study cohort prior to excluding patients who did not have echocardiographic parameters of diastolic function assessed during follow-up (Supplementary Materials Table S1).

Therapy with each of ASA and mavacamten was associated with a significant reduction in LVOT gradient over 5 months of observation (*p* < 0.001) (Table 2). There was no difference between the baseline LVOT gradient (78.2 versus 100.0 mmHg, *p* = 0.11) and follow-up LVOT gradients at 5 months (15.0 versus 23.0, *p* = 0.16) between the ASA- and mavacamten-treated groups, respectively.

**Figure 2:**
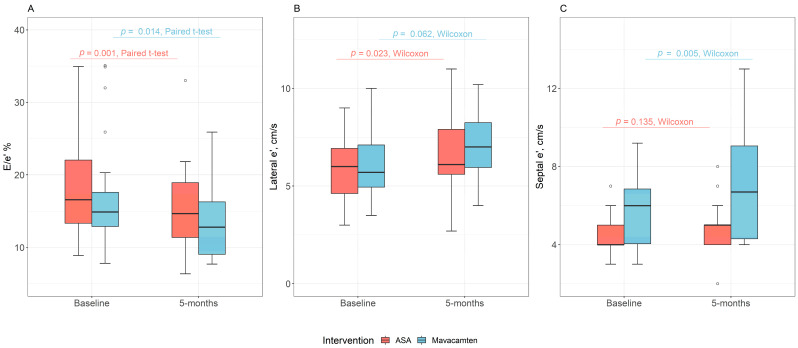
Comparison of baseline and 5-month * outcomes between alcohol septal ablation and mavacamten for (**A**) average E/e’, (**B**) lateral e’, and (**C**) septal e’. * The 5-month endpoint was an approximation with an average of 5.1 months and an interquartile range of 3.5–6.0 months.

In the ASA group, 5 months after ASA, there was an increase in the lateral E’ tissue velocity (6.0 versus 6.1, *p* = 0.02), whereas the septal E’ tissue velocity trended up (4.0 versus 5.0, *p* = 0.14). Over this period, the lateral E/e’ (16.7 versus 12.2, *p* < 0.001), septal E/e’ (20.0 versus 18.0, *p* = 0.02), and average E/e’ (18.6 versus 15.3, *p* < 0.001) values were reduced (Table 2, Figure 2). Average E/A was reduced over the 5-month observation period (0.9 versus 0.8, *p* = 0.043). LA reservoir strain trended towards improvement 5 months after ASA (*p* = 0.05). There was no significant difference in mitral inflow velocity or LA volume index at baseline when compared to 5 months following therapy for patients who underwent ASA (*p* > 0.05 for all) (Table 2).

Similarly, mavacamten treatment was associated with changes in multiple parameters of diastolic function after 5 months of therapy (Table 2). There was an increase in septal E’ (6.0 versus 6.7, *p* = 0.005) with a trend towards improvement in lateral E’ (5.7 versus 7.0, *p* = 0.06) with mavacamten (Table 2, Figure 2). There was a reduction in each of septal E/e’ (16.3 versus 13.1, *p* = 0.03), lateral E/e’ (14.3 versus 11.3, *p* = 0.03), and average E/e (17.4 versus 13.5, *p* = 0.01) (Table 2, Figure 2). Mavacamten therapy was also associated with an improvement in the LA volume index (45.6 versus 34.5, *p* < 0.001). There was no difference in LA reservoir strain with mavacamten treatment. Additionally, there was no change in mitral E or E/A when comparing baseline to 5-month follow-up among patients who were treated with mavacamten (Table 2).

Mitral inflow velocities (E) and E/e’ values (lateral, septal, and average) were not different between the ASA and mavacamten arms at baseline or after 5-month follow-up. The ratio of passive versus active mitral filling velocity (E/A) was similar at baseline and greater in the mavacamten arm compared to ASA at 5-month follow-up (0.8 versus 1.1, *p* = 0.001). LA volume index was greater in the mavacamten arm at baseline (36.2 versus 45.6, *p* = 0.039); however, no difference was seen at 5-month follow up (35.0 versus 34.5, *p* = 0.90) (Table 2).

## 4. Discussion

Cardiac myosin inhibitors (CMIs) have emerged as a tool in the management of obstructive HCM. CMIs have been shown to not only reduce myocardial contractility and outflow tract obstruction, but emerging evidence finds that they are also associated with improvements in diastolic function [22,24,27,28]. In this study, we compare mavacamten to ASA to examine how each of these approaches modifies echocardiographic parameters of diastolic function. Our observations find that ASA and mavacamten yield comparable improvements in LVOT obstruction and echocardiographic parameters of diastolic function.

Specifically, we observe that both ASA and mavacamten are associated with significant improvements in mitral annular tissue velocity (e’) and left-sided filling pressures as determined by E/e’ after 5 months. With mavacamten both septal and lateral e’ improved over time; however, only lateral e’ improved to meet the threshold of statistical significance with ASA. Interpretation of septal e’ velocities should be tempered by the knowledge that this may not be a reliable measure of diastolic function in patients who have undergone ASA [29]. It has been proposed that direct injury of the basal septum by ASA results in tissue fibrosis and thereby diminishes tissue velocities and the sensitivity of septal e’ as a measure of diastolic function.

In addition to diastolic function, those in the mavacamten group showed a statistically significant decrease in LAVI at 5-month follow up. Given the small size of the study, this finding would require further validation in a larger cohort.

Differences in clinical characteristics between our study groups are present and thereby limit the reliability of direct comparison. The study groups were comprised of individuals with similar BMIs and sex-ratios; however, patients in the ASA arm were older and more likely to have prior tobacco exposure. Individuals in the ASA cohort were also more likely to have been treated with non-dihydropyridine CCB and/or disopyramide compared to their mavacamten-treated counterparts. The differences observed may reflect an overall older and sicker population in the ASA arm. Indeed, a larger fraction of patients in the ASA arm reported functional limitations that met NYHA Class III criteria. Nevertheless, despite these clinical differences, there was no difference in LVOT obstruction, mitral inflow velocity (E), lateral and septal e’ values, and left-sided filling pressures as determined by E/e’ at baseline.

Lastly, the small study size, single-center focus, and retrospective nature limit the generalizability of these observations. Short-term follow-up impedes determination of long-term features of this comparison. Large multi-center trials with extended follow-up will be required to address these questions. Furthermore, longer-term follow-up may show more significant improvements in diastolic function parameters, including LAVI. At this time, follow-up was likely too short to see improvement in all diastolic parameters.

We previously reported that ASA and mavacamten yielded comparable performance characteristics with respect to hemodynamic parameters (LVOT, ejection fraction, and mitral regurgitation) and associated improvement in patient functional status (NYHA class) [23]. This is the first study of its kind to compare improvements in echocardiographic parameters of diastolic function between patients who underwent ASA against those who were treated with mavacamten. Importantly, these data suggest that relief of outflow tract obstruction, independent of therapeutic approach, may be sufficient to improve hemodynamic parameters that define diastolic function. Undoubtedly, larger studies are required to further characterize the relative efficacy of the two therapeutic modalities.

## Figures and Tables

**Table 1:** Study cohort demographics, comorbidities, and medications.

Characteristic, No. (%)	Alcohol Septal Ablation	Mavacamten	*p*-Value ^1^
	*n* = 22	*n* = 23	
Age, years, mean (SD)	76.0 (9.0)	61.3 (12.7)	<0.001
Females	17 (77.3)	13 (56.5)	0.140
BMI, kg/m^2^, mean (SD)	31.4 (8.3)	31.9 (6.4)	0.829
Tobacco use	17 (77.3)	3 (13.0)	<0.001
Atrial Fibrillation	3 (13.6)	4 (17.4)	1
Hypertension	19 (86.4)	15 (65.2)	0.099
Diabetes	3 (13.6)	6 (26.1)	0.459
ICD	3 (13.6)	8 (34.8)	0.099
Beta blockers pre-therapy	20 (90.9)	21 (91.3)	1
Non-dihydropyridine CCB pre-therapy	8 (36.4)	0 (0)	0.002
Disopyramide	6 (27.3)	1 (4.3)	0.047
NYHA			0.007
II	6 (28.6)	16 (69.6)	
III	15 (71.4)	7 (30.4)	

^1^ Two-sample *t*-test, Chi-squared tests, or Fisher’s exact tests as appropriate.

**Table 2:** Comparison of baseline and 5-month outcomes between alcohol septal ablation and mavacamten for LVOT gradient, diastolic function parameters, LA volume index, and LA reservoir strain.

Characteristics, Median (IQR)	Baseline			5-Months *			**Pairwise *p*-Value for** **Baseline vs. 5-MONTHS ^2^**	
	ASA	Mavacamten	*p*-Value ^1^	ASA	Mavacamten	*p*-Value ^1^	**ASA**	**Mavacamten**
LVOT gradient, mmHg	78.2 (57.8–102.0)	100.0 (64.0–128.1)	0.114	15.0 (7.0–41.0)	23.0 (14.4–62.5)	0.159	<0.001	<0.001
Mitral E, m/s	1.0 (0.7–1.1)	0.9 (0.7–1.2)	0.900	0.8 (0.6–0.9)	0.9 (0.7–1.0)	0.583	0.117	0.448
Mitral E/A	0.9 (0.7–1.1)	1.0 (0.7–1.4)	0.206	0.8 (0.7–0.9)	1.1 (0.8–1.2)	0.001	0.043	0.882
Septal E’, cm/s	4.0 (4.0–5.0)	6.0 (4.0–7.0)	0.027	5.0 (4.0–5.0)	6.7 (4.1–9.1)	0.020	0.135	0.005
Lateral E’, cm/s	6.0 (4.5–7.0)	5.7 (4.9–7.2)	0.793	6.1 (5.6–7.9)	7.0 (5.9–8.5)	0.430	0.023	0.062
Average E/e’, mean ± SD	18.6 ± 7.3	17.4 ± 7.5	0.595	15.3 ± 6.1	13.5 ± 5.0	0.281	0.001	0.014
E/E’ septal	20.0 (16.7–25.0)	16.3 (13.0–20.0)	0.046	18.0 (14.0–22.5)	13.1 (9.0–19.5)	0.040	0.023	0.030
E/E’ lateral	16.7 (11.7–22.5)	14.3 (11.5–20.0)	0.586	12.2 (9.6–18.4)	11.3 (9.1–16.0)	0.613	<0.001	0.032
LA volume index, mL/m^2^, mean ± SD	36.2 ± 13.4	45.6 ± 15.2	0.039	35.0 ± 15.6	34.5 ± 9.6	0.898	0.510	<0.001
LA reservoir strain, %, mean ± SD	31.3 ± 9.6	28.2 ± 8.8	0.267	25.4 ± 10.5	31.3 ± 13.7	0.124	0.047	0.298

^1^ Two-sample *t*-test or Wilcoxon tests as appropriate; ^2^ Paired *t*-test or Wilcoxon signed rank test as appropriate; IQR = interquartile range; SD = standard deviation; ASA = alcohol septal ablation; ASA, *n* = 22; mavacamten, *n* = 23. * The 5-month endpoint was an approximation with an average of 5.1 months and an interquartile range of 3.5–6.0 months.

## Data Availability

Primary data can be made available upon reasonable request.

## Supplementary Materials

The following supporting information can be downloaded at https://www.mdpi.com/article/10.3390/jcdd13010016/s1, Table S1: Demographics, comorbidities, and medications of the sample population prior to excluding patients who did not have echocardiographic follow-up of diastolic function parameters.

## Abbreviations

The following abbreviations are used in this manuscript:

HCM	Hypertrophic cardiomyopathy
ASA	Alcohol septal ablation
LA	Left atrium
LVOT	Left-ventricular outflow tract
SRTs	Septal reduction therapies
IVS	Interventricular septum
REMS	Risk Evaluation and Mitigation Strategy
LVEF	Left-ventricular ejection fraction
NYHA	New York Heart Association
ASE	American Society of Echocardiography
BMP	Body mass index
CMIs	Cardiac myosin inhibitors
LAVI	Left-atrial volume index
