# Supplementary material for: Comparison of Diastolic Function Parameters After Alcohol Septal Ablation and Mavacamten Therapy in Obstructive Hypertrophic Cardiomyopathy
Source: J Cardiovasc Dev Dis. 2025 Dec 29;13(1):16. doi: 10.3390/jcdd13010016 (PMC12842295; doi:10.3390/jcdd13010016)
Supplement: Supplementary file 1 [file jcdd-13-00016-s001.zip › jcdd-4051383-supplementary.pdf]

Table S1. Demographics, comorbidities, and medications of the sample population prior to excluding patients who did not have echocardiographic follow-up of diastolic function parameters.

Characteristic, no. (%)	Alcohol Septal Ablation	Mavacamten	P-value[1]
	N = 58	N = 48	
Age, years, mean (SD)	73.6 (8.7)	62.1 (12.9)	<0.001
Females	39 (67.2)	27 (56.2)	0.25
BMI, kg/m^2, mean (SD)	31.9 (7.7)	31.5 (6.6)	0.77
Tobacco use	34 (58.6)	12 (25.0)	<0.001
Atrial Fibrillation	11 (19.0)	7 (14.6)	0.58
Hypertension	44 (75.9)	33 (68.8)	0.41
Diabetes	9 (15.5)	7 (14.6)	0.89
ICD	11 (19.0)	11 (22.9)	0.62
Beta blockers pre-therapy	53 (91.4)	38 (79.2)	0.07
Non-dihydropyridine CCB pre-therapy	16 (27.6)	9 (18.8)	0.29
Disopyramide	23 (39.7)	5 (10.4)	<0.001
NYHA			<0.001
II	22 (38.6)	36 (76.6)	
III	35 (61.4)	11 (23.4)	

[1]Chi-squared tests or two-sample t-Test as appropriate.
